# Contractant behaviour in the pandemic: A real-world survey

**DOI:** 10.1177/20555636221117774

**Published:** 2022-06

**Authors:** Charles Haward Soper

**Affiliations:** 4488 University of Leicester, Leicester, UK

**Keywords:** Contract law, contract management, empirical research

## Abstract

Entirely predictably the Covid pandemic caused material problems in the contracting world, issues such as delivery, payment, and liability, suddenly achieving an undesired prominence. As an empirical researcher, it struck me that I might test my overarching belief that the only valid interest in contract is the performance interest by asking experienced contract professionals how contractants had behaved in the pandemic. During the pandemic, these professionals encountered disputes, a deeper examination of contract provisions, claims, and debates on internal processes. All of which stabilised over time as pragmatic, performance focussed management took the reins. Respondents reacted professionally to events, working to keep contracts alive and reconfigure relationships using a mixture of the formal and the informal, in governance and in the commercial relationship. If contracts did not quite work, respondents engaged, adjusted and trimmed. The same was true for processes – If they did not quite work ‘work arounds’ were created. The paper considers two plausible commercial scenarios in which current contract law might allow an opportunist player to escape from the pandemic bargains made, and argues that contract law should support deal-makers and deter such opportunism.

## Introduction

On 15 July 2020, I presented an ‘Ask the Expert’ webinar in which participants discussed the reaction of professionals responding to a dispute between a facilities management contractor and an NHS Trust.^1^ One attendee asked how the pandemic was affecting the way people managed contracts, which provided a platform for an intriguing piece of research, a short chapter in Stewart Macaulay's Grand Narrative, a ‘meta-narrative’ based on ‘an inner connection between events … a succession of social systems, the gradual development of social conditions …’.^2^ Earlier empirical survey work in Soper, Commercial Expectations and Cooperation in Symbiotic Contracts - a Legal and Empirical Analysis, allowed me to conclude that ‘… This claim, that the performance interest is the only pure contractual interest’^3^ means, as Sir Kim Lewison elegantly puts it – ‘both parties to a contract are taken to contract on the footing that they wish the contract to be performed’^4^ or, similarly, as Sir John Mummery has written ‘Promises are usually made … in the expectation that they will be kept rather than broken.’^5^ The claim is, again, supported by survey responses. It is also reflected in responses to other researchers such as Russell Weintraub who found that Corporate Counsel strongly supports expectation measure damages (damages that put the innocent party in the position they would have been in had the contract been performed).^6^ The survey was intended to explore whether the pandemic had dented this core interest in performance.

Five aspects of contract reality were explored:

- Whether behaviour had changed in the pandemic and if so how?
- How contracting and governance processes had performed?
- Whether respondents had experienced disputes and how disputes were managed?
- How the pandemic would affect risk analysis and appetite for risk in the future?
- Whether the pandemic will change the way contracts are managed?

Respondents were experienced contract managers or procurement experts who understand in broad terms the relevant background^7^ to modern contracts, whose ‘lived experience’ can provide a sense of the effects of the pandemic on the behaviour of contracting parties, who possess some underlying commercial common sense, and who can be fairly described as among the notional reasonable people referred to by Lord Neuberger in one key contract case.^8^

## Respondent sample and demographics

The research is classically qualitative, digging into and around the lived experience of respondents, leveraging my professional familiarity with the setting.^9^ Such research sampling requires a realist, judgmental,^10^ purposive approach to finding an ‘appropriate’ population, and deployment of ‘special expertise or knowledge’,^11^ and even opportunism and personal contacts.^12^ In such research, the researcher's judgment is, as Colin Robson suggests, the leading selection criterion.^13^ Having held senior commercial/contracting/legal roles in major multi-nationals, such as GEC, Siemens, Shell and Alstom, spending over 30 years in global commercial environments, in shipbuilding, oil industry fabrication, power, defence and marine, nuclear fuel reprocessing, airport baggage handling, automated warehousing, oil and gas, compressor manufacture, and gas and steam turbine manufacture I am qualified to use my own judgement.^14^

Non-random samples are the norm in similar studies, one researcher noting that ‘virtually all samples used in I-O psychology are convenience’^15^ and Alan Bryman that this is ‘… typical in management and business studies’.^16^ A random sample, using a defined population, selecting a representative sample, is not practically possible for contract managers and commercial enterprises are generally unable or unwilling to provide population data to researchers.^17^ Colin Robson notes that:

The exigencies of carrying out real world studies can mean that the requirements for representative sampling are very difficult, if not impossible, to fulfil.^18^

Evocatively, Miles and Huberman observe:

social processes have a logic and a coherence that random sampling can reduce to uninterpretable sawdust^19^

The (132) respondents have in common experience of contracting, and they constitute a variegated, diverse, global sample with profound, wide-ranging experience and background. As a control respondents were asked the same question asked in earlier research (Table 1).

**Table 1.** What does cooperation mean?

What does cooperation mean in contract management?… You may select more than one				
	2015–2016 483 respondents	2021 132 respondents 36 F (27.3%) 94 M (71.2%) 2 did not say		
Working together, sharing responsibility for outcomes, putting aside party interests, working towards a joint or mutual goal in a relationship underpinned by mutual trust.….	277 57.3%	85 64%	20F 55%	65M 69%
Each party acting reasonably, and objectively, not opportunistically, when problems occur, being flexible with solutions where the problem is not fundamental.…	340 70.4%	98 74%	30F 83%	66M 70%
Flexible decision making and change management especially in administering changes and variations.…	144 29.8%	57 43%	14F 39%	41M 44%
Use of the same programme management tools and management systems, good day to day coordination of activities.…	91 18.8%	26 20%	6F 17%	19M 20%
Other	24 5%		5 4%	

There is some variation in the raw numbers, but the pattern remains the same; respondents overwhelmingly chose mutuality (the first option) or active cooperation.

Denzin coined the term ‘triangulation’ for carrying out studies in different locations, using multiple theories, researchers, or data technologies, different sources, collection methods, quota samples, age and gender, and datatypes to help ascertain how far one might generalise from a non-random sample.^20^ Demographic data was collected to enable comparison/triangulation between subsamples to determine the robustness and consistency of the data. Of those responding 71% are male, 27% female with two who preferred not to say. Eighteen percent operate in the public sector, 70% in the private sector, 1% in the third sector, 4% in education, 6% mixed and 2% other. Professionally, (rounded) 18% are project managers, 10% technical/engineers, 54% contracting/procurement specialists, 24% commercial specialists, 22% lawyers, 2% facilities managers and 11% others. 52% emanate from an English Common Law legal culture, 7% from US Common Law, 14% civil law, 1% China, 6% mixed (Scotland Philippines or South Africa), 19% other and 2% emerging jurisdictions. Because it might show that as people move up the greasy pole their outlook might become more or less cooperative data was collected on seniority finding that 39% were executive managers or directors, 10% general managers, 21% contract or project managers, 17% managers and 13% other. To determine whether attitudes would change with portfolio value data on this was also collected; 11% had portfolios of up to $1M, 21% between $1M and $10M, 24% between $10M and $100M, 22% between $100M and 1Bn and 22% more than $1Bn. As less experienced respondents might be more inclined to manage in ‘tell’ mode and use formal contractual mechanisms more than those with significant experience longevity data was also collected, finding that 6% have 1–5 years of experience, 16% have 6–10, 33% have 11–20 and 44% over 20. Sixty seven percent have expertise in projects or infrastructure, 35% in facilities management, 20% in R&D, 53% in consulting, 41% in IT/Telecoms, 39% in long-term maintenance, 14% outsourcing, 2% franchising, 49% long-term supply and 51% equipment supply.

## Survey and interview design

The basic collection tool was an online survey taking into account multiple requirements:

- The lived experience was key. Questions were, therefore, realistic.
- The conversation was with an elite. The survey was, therefore, complex, employing open questions.
- The availability of online survey tools, easy to distribute by email or social media, made an online survey an easy option despite the risk, realised, that a lot of data would be returned for analysis. This survey used the Bristol Online Survey tool (www.onlinesurveys.ac.uk/) which makes design easy.

## Survey results

### Contracting behaviour in the pandemic

The survey opened with an open and a scaled question. The open question was ‘Has behaviour in contract changed during the pandemic? Is it better or worse or much the same? Can you briefly describe any changes (without identifying any person or company)’ and the scale that followed asked how behaviour had changed; was it ‘Significantly better’ (3%), ‘Better’ (25%), ‘Much the same’ (39%), ‘Worse’ (19%), or ‘Significantly Worse’ (7%), with 7.5% reporting that it varies. A snapshot of gendered responses provides an interesting picture with female respondents as likely to report better behaviour but apparently much more likely to report worse.


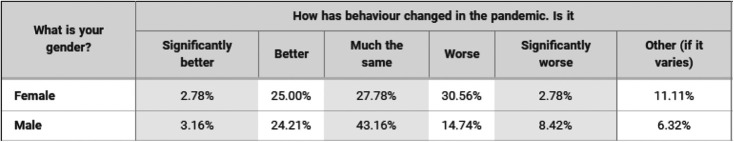


This seems striking, but it is not *statistically* significant.^21^ In earlier research, I found that male and female respondents differed little in their managerial approaches.^22^ One possibility is that, given that male respondents outnumber females by 3 to 1, females might encounter less collaborative behaviour from males than males do.^23^ It's only one possibility but it seems to be an area worth some research.^24^ There appears not to be a hierarchical explanation as female respondents tended to have more experience and 53% held the two ‘highest’ titles, compared to 50% of the sample.

‘We are all hurting’ was the haunting comment of one respondent and others agreed with this sentiment:

- people have found a need for greater human contact and there is a sense of **all being in this together**,
- there seems to be a higher level of aspiration to be collaborative,
- everyone is more cooperative and agile,
- commercials have gone out the window a bit in favour of relationships,
- we preferred to take an adult-to-adult approach.

The voice of the ‘much the same’ group came through clearly, one saying that with ‘contracts under acute pandemic-driven pressures, behaviours have had to respond’, and one hard-bitten soul saying – ‘Much the same [client] in cost denial’. Others said that behaviour was ‘often reflective of the quality of the pre existing relationship’, another reported contractors trying to ‘protect/retain their business … even at the cost of reduced profit margins … or volumes’. Another said that it was business as usual ‘… innovated round the challenges’.

One who reported mixed behaviour contrasted the behaviour of UK suppliers unfavourably with overseas suppliers, another saying ‘… some working stricter and other carrying on as usual’. Another was analytical – ‘This environment has become an interesting test of how suppliers view you – for some … it's strictly quid pro quo, others … value relationship more’. Another said, ‘Some companies take pragmatic approach for example just request extension of time but some request time and cost which is difficult to agree …’.

Of those reporting worse behaviour, 17 commented including:

- easier now for people to hide behind emails,
- clients refusing to accept ‘that the pandemic has caused/continues to cause delays in equipment manufacturing’,
- ‘blanket “cos covid” excuse’,
- using the pandemic as a bandwagon for a greater number of spurious claims,
- pressure to continue business despite the risk to employees and against the government ‘stay at home’ advice,
- issues around late delivery have become increasingly contentious which has necessitated all parties to behave in a more adversarial manner. This is not beneficial to long-term relationships,
- suppliers are seeking to rely upon Force Majeure provisions with varying degrees of success.

None of this seems surprising and as we shall see little was irresoluble.

Of those reporting better behaviour, eight commented:

- in a certain way it brings people a bit closer,
- we have all had to make difficult choices,
- others have been excellent, dealing with any required changes pragmatically and without recourse to contract terms and building on the goodwill.

Contract drafting issues were mentioned by 26, 14 discussing force majeure clauses, some reporting that they had accepted that the pandemic was a force majeure event, others that – ‘it is still being argued that a global pandemic and associate global government restrictions do not meet the definition for Force Majeure’. Another reported that despite acceptance of the fact of a force majeure event ‘… it should be factored into pricing/scheduling, but at the same time are not willing to accept delays or increased costs’ – a desire for the best of both worlds. Others reported pragmatism – ‘we have had to add a “Pandemic” clause’. In 2020, WorldCC (*Managing Contracts under Covid-19: What have we learnt?*) reported that ‘The greatest difficulties have been encountered in finding and reviewing Force Majeure clauses, undertaking analysis of key performance terms and responding to management questions …’.

One effect of the pandemic has, of course, been an increase in remote working on which there was some divergence in views, several discussing work slowing down ‘Everything is taking twice as long to complete’. Others discussed ‘fragmented’ negotiations – ‘negotiation won't in most cases proceed in one piece’, ‘Lack of face-to-face time’ or ‘more time consuming due to online negotiations’ and one that ‘my feeling is that this does reduce the level of “closeness” in the relationship and makes it a bit harder to build trust’. Another thought behaviour worsened because ‘it is more complicated to bring the right people together’. Others reported the opposite ‘Faster discussions to collaborate, less competitive behavior impacting negotiations’, ‘increased urgency and faster decisions on priority projects’, ‘online meetings have made leaps and bounds for the obvious reasons’, ‘behaviour may have speeded up’, or even ‘became better, more efficient’. It may be that remote working tends to confound the issue; previous research has found that teleworkers are more satisfied with work than office-based workers.^25^

The National Residential Landlords Association surveyed its members. A total of 60% of the 4566 respondents reported a loss of income, with only 4% having determined to take legal action.^26^ They reported:

one of the tenants is carrying out both gardening and additional cleaning in lieu of rent,

I have decided to work with my tenants in order to keep them housed and agree a payment plan for rent arrears

Ultimately, I would prefer to help her through anyway

Landlords were asked whether they had been approached for rent reductions, deferrals, or holidays and 44% recorded that they had. Over 90% reported that they had responded positively to such requests, one saying ‘… as **we are all in this together’**, and later in the pandemic Tom Witherow reported (https://www.thisismoney.co.uk/money/markets/article-9785343/Landlords-war-1-6bn-unpaid-rents.html) ‘claims that one in seven commercial tenants have ignored approaches by their landlord to avoid entering talks to agree repayment plans’; indicating engagement in the vast majority of cases. Post-pandemic sharp rise recorded in landlords seeking to recover rental debt | Property Reporter


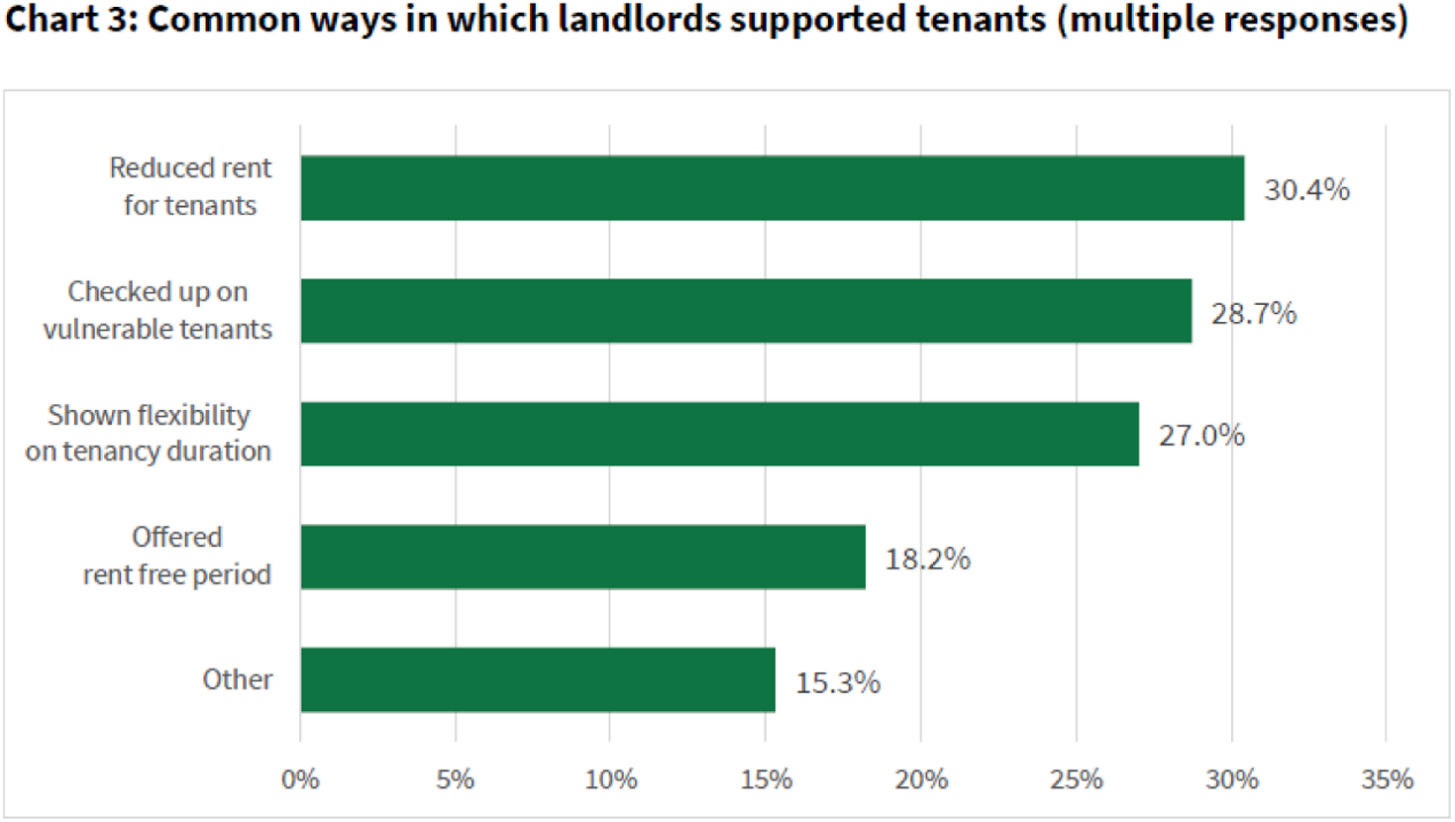


There are, however, other reports of increases in illegal evictions during the pandemic.^27^

### Were your processes adequate?

One former company used the following practical (and often ignored) meta-guidance on processes:

No procedure will be of any use if it is performed mechanically without a continuing sharp focus on the circumstances to which the policy is directed.

Respondents were asked whether processes had worked, whether adjustments had been made (and how they were managed) and whether the respondent had sufficient authority to deal with adjustments. One commented, rightly, that ‘A well-written governance process should deal with any changes’. It was still something of a surprise that processes were found to work (albeit often with workarounds, reported by 40% for governance and 36% for contracting) by the overwhelming majority, a mere 7–8% reporting that they had worked badly or very badly. As a former owner (and the e-process designer) of a very tough and complex contracting process for Siemens’ Oil and Gas Division I know that people bellyache about processes, so it is interesting to find that in extremis key processes worked.


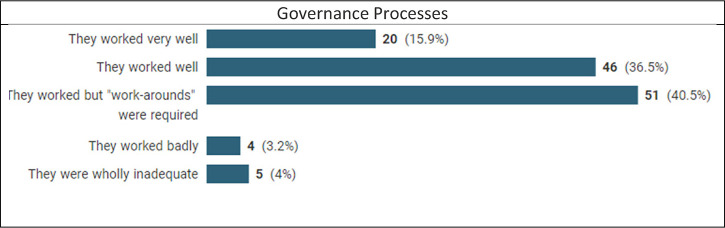



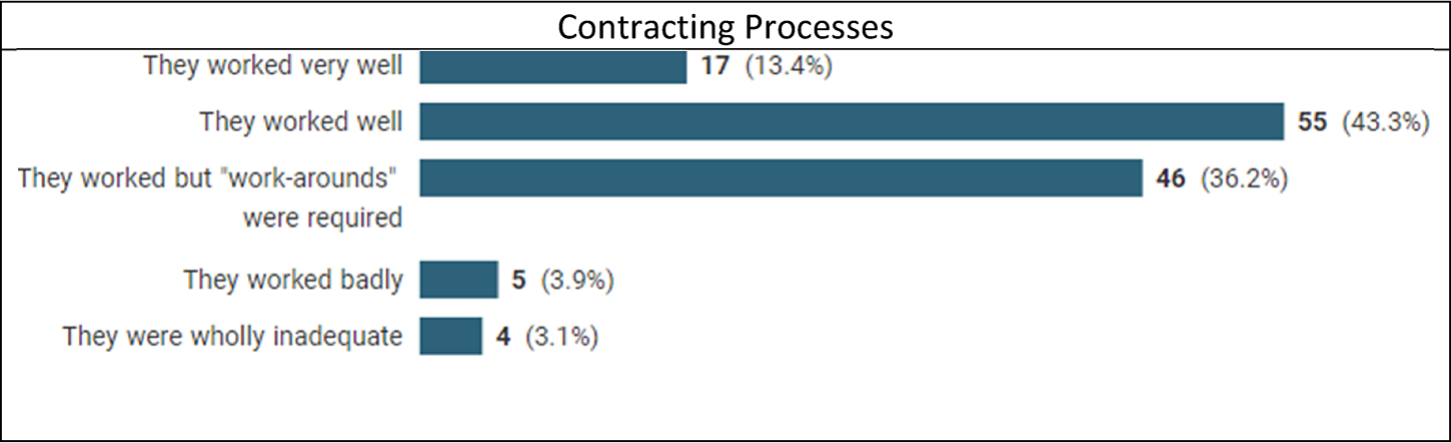


Seven commented on workarounds which included waivers of inspection requirements, advance payments, waiving penalties, and retro-fitting Force Majeure clauses. One mentioned compliance concerns, and nine commented on speed and streamlining:

- Contracting was more agile and focus on outcomes aided by a desire for people to leverage the co-operation.
- The cumbersome regime of stage gate reviews … were immediately consigned to the process graveyard and a more dynamic approach evolved which demanded availability and decision-making from senior management/budget holders.
- … speed was an issue where procedures had to be followed in spirit rather than the letter.

Fourteen mentioned flexibility or collaboration:

- … first contracts we were essentially developing new policies on the fly … governance permitted this fairly well.
- due diligence appears more relaxed.
- Both parties, in most cases, worked on the challenges … in a collaborative manner.

One interviewee commented that ‘Sometimes more junior staff would change things and get over-ridden …’

Over 80% said that they had sufficient authority all or most of the time to deal with the necessary adjustments to contracts, including, in one case, the ability to increase costs by up to 10%. Other parties were reported to have been ‘awkward’ or ‘extremely challenging’ or even ‘in denial’. There were occasional niggles about being overruled or ‘overkill’, but some recorded that they had simply required broader consultation. Some reported that contractual relief had not posed any compliance or authority issues. This suggests that things may have changed since 1963 when Stewart Macaulay suggested that resolving disputes had more to do with where corporate power lay than in the formal process.^28^

That UK Government procurement processes were inadequate, circumnavigated or ignored in material respects, particularly of the large-scale pandemic requirement for Personal Protective Equipment is notorious.^29^ The National Audit Office recommended that in future ‘… government identifies and manages potential conflicts of interest and bias earlier in the procurement process’. The overall picture is, however, that most processes worked well, and that when workarounds were instituted, they worked. It is arguable that in this compliance area people paid attention to the main goal, performance, relegating black-letter process compliance to its rightful position, and making the appropriate adjustments to processes and/or obligations.

It seems likely that corporations design their contracting and governance processes allowing flexibility sufficient to cover even a major disruptor without the need for process revision. Respondents used this inherent flexibility and did not treat processes as black letter instructions which ensured that business contracting could continue. This is a good indicator that contracts are not managed as lithoglyphs, but as business instruments.

### Did you experience disputes?

Respondents were asked whether the pandemic had caused disputes, whether ‘serious’ or ‘not so serious’, whether they were resolved or not and if not, how they would be resolved.

Only 34% or 47% experienced serious disputes, with one experiencing one and four experiencing many. Thirty seven percent or 28.5% experienced a few. Twenty had settled all disputes. Of those 27 outstanding 22 reported, in open questions, that settlement would be by negotiation, or through some flexibility. Those negotiating talked of ‘Attrition and leverage’ in one case and in another ‘Trade off deals for future work’, hardly unfamiliar ground.

Three thought that disputes would end up in Court, two in mediation, two arbitration and two adjudication. One said they would resolve ‘By punitive punishment when that's possible’ (!).

Unsurprisingly, more, 57% or 75%, experienced minor, less serious disputes. Just under 10% or 12% experienced many, 58% experienced a few and five reported only one. Of those disputes just over half were wholly resolved, just over one-third reporting a few unresolved and 12% reporting many unresolved. One respondent thought that unresolved disputes would end up in Court, due to ‘Public Procurement’, with 23 considering that negotiation or flexibility (‘Amicable settlement meetings’) would settle disputes. One said, ‘These are not the times to be overly “legal”’ and another mentioned ‘some local friction … resolved via relationship management’. Another referred to ‘Just ironing out governance and ways of working … with the supplier’. Of those in more difficulty, one described a supplier taking ‘a take it or leave it attitude to price increase’ and that they had ‘little option but to take it’. One consultancy described ‘panic’ by ‘businesses on the verge of bankruptcy’ but ‘[we] want clients to survive’. Another took a pragmatic, relationship focussed view:

We will simply reward suppliers who supportive of our business during the pandemic and potentially do less business with those who were not …

Beale and Dugdale explained that one reason for the avoidance of lawyers and legal remedies is that they are perceived to be inflexible.^30^ This was not reflected in the comment, in fact one respondent referred to ‘Legal took the authority generally’ when discussing process management. Macaulay was more diplomatic, describing in-house Counsel as favouring ‘neat and tidy’ formality.^31^ The only ‘anti-lawyer’ comment referred to one lawyer ‘drumming up business’ based on taking advantage of the pandemic. The overall picture appears to be that from defensive positioning at the start of the pandemic, in which force majeure, and extensions of time were in issue, parties have moved to normalise matters by resolving disputes and working normally or more collaboratively. Contractants consider that they are ‘better off settling their disputes themselves, away from the courts’.^32^ The UK Government ‘advised parties to consider “equitable adjustment or accommodation” … resolution “through negotiation, an early neutral evaluation or mediation’.” Those still in the trenches are a small minority.

### Has the attitude to or appetite for risk changed?

Asking a closed tick-box question to identify how risk management and analysis might change resulted in these responses:


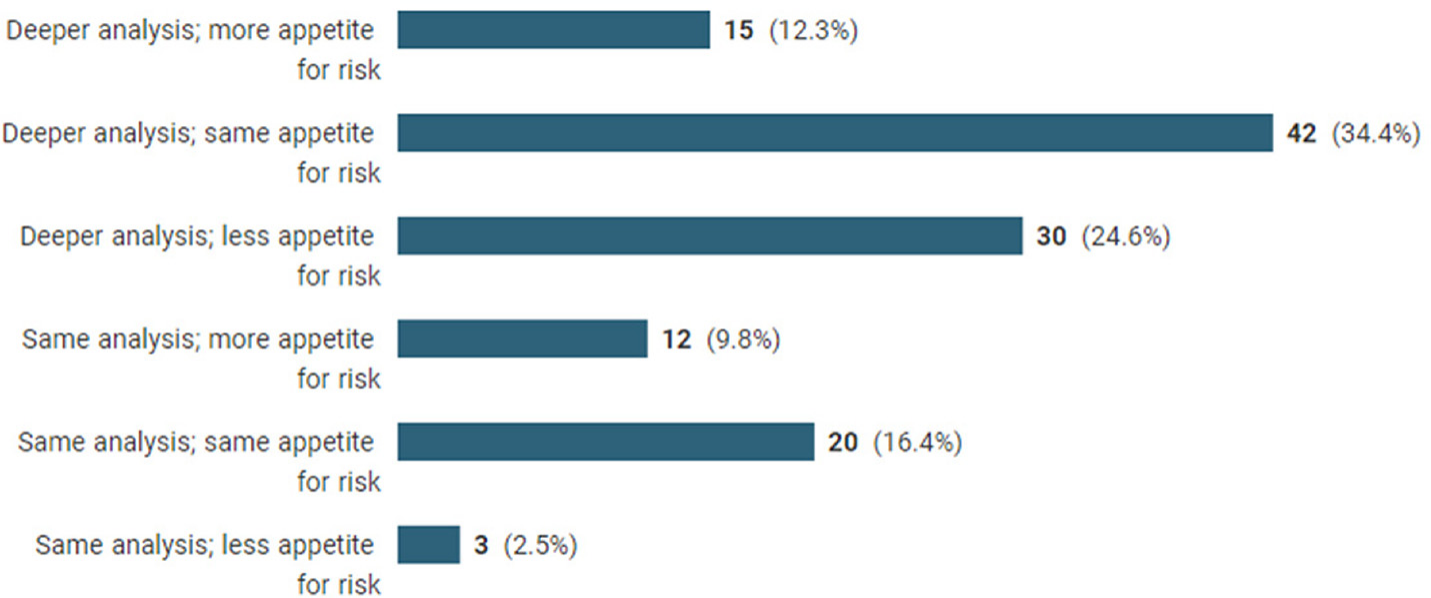


Over 70% of respondents say that risk analysis will deepen. It may be that many of these have identified gaps in their risk analysis processes, and from other responses, it can be inferred that pandemic management and the drafting of ‘force majeure’ clauses have been popular topics of internal debate.^33^ Only 16% report that they will change neither analysis nor approach to risk, from which it can be inferred that almost everyone must have reviewed this issue, as even those who elected for no change are highly likely to have considered the point. One start-up reported that their process review had resulted in a quicker, cheaper and more effective risk analysis process.

Around 50% of companies retain their current risk appetite, and two-thirds will undertake deeper analysis. Twenty seven percent have less appetite for risk, but a mere 10% of those will not undertake deeper risk analysis. Twenty two percent of respondents identify an increased appetite for risk of which almost half said they would not undertake deeper risk analysis (‘just more investigation into covid specific risks’). One hundred and fifteen responses to the question ‘Has the pandemic affected your organisation's attitude to or appetite for risk?’were received. Few discussed the detail, although one was quite interesting ‘Better consideration of trade deals … insight into supply chain resilience. Sub-tier market knowledge for raw materials and alternative products’. Several mentioned the need to cater more clearly for force majeure – ‘a bit more emphasis on ensuring that pandemics are included as a force majeure event’. One said that it meant ‘more focus on the importance of due diligence’. The comment included the dry observation that ‘stakeholders realise that pandemics do happen’, and that the pandemic has ‘highlighted how contracts need to be more flexibly structured to allow for significant changes in demand’ and the need for ‘increasing project level contingencies’, with consideration given to ‘greater tendency towards risk mitigation … rather than risk allocation’, which reflects the ‘**we’re all in this together**’ attitude. One, more one-sided respondent had become ‘more risk averse and eager to include contractual parameters to protect our positions in similar future scenarios’. The IACCM's (now World CC) pandemic report (business impact of coronavirus emergence) said that collaborative relationships imply a more balanced approach to risk allocation terms and a greater focus on provisions which assist identification and management of risk. One respondent noted ‘All parties have recognised that the market activity has changed and that there needs to be a review of how risk is shared’ and another:

There is a balance to be struck between us assuming risk that really should belong to the customer, but we are willing to share some of it. We hope this will pay off both in goodwill and allowing our customers to actually remain in business!

Discussing how the pandemic would affect contract management in the future 28 respondents mentioned risk management, ranging from the specific, ‘We will take the threat of pandemics more seriously’, through ‘Tighter terms regarding safe working conditions and travel for work’ to more systematic considerations, ‘Better insight into supply chain resilience’ and ‘There is definitely a thirst for a more linear approach to contract risk …’.

### How will the pandemic affect the way contracts are managed?

This was an open, optional question and respondents took time to consider their thoughts. One optimist hoped:

… that we will become more kind and trusting, given our shared experience, that will enable better collaboration and relational contracting

Eighteen thought that little would change, one saying it was a little like wilful misconduct ‘after Deepwater Horizon’, another that ‘ultimately these scenarios were catered for, but only in a broad sense, another that ‘already the old regime is starting exert its authority and slow decision making’. Two thought it too early to say, one observing:

not sure it is prudent to attempt to mitigate something like this in the future … I suspect many will try, but then realize it is not particularly practical to manage something that might look very much like a ‘one-off’ a few years from now

Twenty-seven mentioned less face-to-face interaction or more IT, one with a little regret – ‘new meetings operate at 80% efficiency … loose the body language, the micro-interactions’. Paul Fisher notes that ‘One of the challenges in negotiating online is that you miss those non-verbal signals when someone is getting frustrated, anxious, angry or showing other emotions’^34^. Another respondent observed that it is – ‘still advisable to meet in person a couple of times, get to know each other's … “tell-tales’,” an interviewee saying ‘trust suffers … you build trust with the client through chemistry, through having a few pints after work’ (which is, from experience, probably a UK respondent), or ‘Harder to decide if you like or trust someone over a video screen’ and another ‘… there's a real problem with maintaining relationships online … you really miss the serendipity that comes from bumping into people in the office. It is easy to become isolated, and even a bit paranoid’. Twenty-seven mentioned improved contract management, including drafting. One said that the pandemic had thrown a spotlight on waste and that ‘contract management will get more focus moving forward’, another that ‘proper contract management is required to understand the full implications of the pandemic’. Another thought that ‘change management will be dramatically streamlined’. Others foresaw ‘Thorough and active management of the contract’ or ‘Increased use of the contract as a living, breathing document’. And, bringing tools and management together one said that:

Thanks to the acceptance of using online communication tools, my impression is that the parties are more in touch than before during the project execution phase. The parties now have more opportunities to tackle small issues before they turn into serious problems.

Interestingly a major Microsoft survey revealed that people want to have their cake and eat it:

over 70 percent of workers want flexible remote work options to continue, while over 65 percent are craving more in-person time … 66 percent of business decision makers are considering redesigning …. The data is clear: extreme flexibility and hybrid work will define the post-pandemic workplace.

One was less polite about the numbers departments – ‘It's far more efficient to actually meet person to person but I fear that the bean counters will just see the travel cost reductions and not the efficiency cost’. Another respondent said ‘all that [travel] budget money had been shifted to the profit column’. Others talked of the benefits of speed with increasing automation.

There were other references to relationship issues, 13 raising this directly, one hoping that the ‘shared experience’ would be reflected in better future relationships and others that ‘Relationship and trust has always been key to effective contract management, but will become even more critical’, the ‘Pandemic has brought a more partnering/working together to resolve approach’, and another that there is ‘More flexibility around supporting clients’. Another thought the pandemic is ‘a shot-in-arm on how important a deeper client–contractor relationship needs to exist’.

## Legal pitfalls and ethical dilemmas

As Sir Kim Lewison notes, contracting parties expect contracts to be performed.^35^ Serious contract players do not make informal or tacit agreements for fun. Deal-makers are right to expect Courts to respect these deals and should not be faced with technical rules lurking in the undergrowth of contract law which undermine them. Remember (see above) that over 80% of respondents considered that they had sufficient authority to deal with contract adjustments. In this section two of these rules are reviewed, arguing that they incentivise undesirable behaviour and fail to respect party autonomy. Those rules are

- the axiomatic approach taken by the Supreme Court to No Oral Modification (NOM) clauses in *Rock Advertising v MWB Business Centres* (‘*Rock*’)^36^ and
- the rule against using subsequent conduct in contract interpretation,^37^ which was criticised by the great Common Law Judge, Lord Steyn saying that – ‘business people simply do not understand such a rule’.^38^

In *Rock* in a ‘radical’^39^ solution, of which Janet O'Sullivan approves, which, nonetheless, says Paul Davies, represents a ‘significant departure from that of other common law jurisdictions’^40^ the Supreme Court made a literal interpretation of a clause saying that ‘All variations to this licence must be agreed, set out in writing and signed on behalf of both parties before they take effect’ invalidating a clear agreed oral variation.

As we know and as described there were immediate pandemic-related problems in supply chain and contract management, leading to disputes, examination of contract provisions, claims, and debates on internal processes, which stabilised over time as management took the reins, driving performance in a pragmatic manner. Respondents reacted professionally to events, working to keep contracts alive and reconfigure relationships. In their ‘Breathing Space’^41^ notes, the British Institute of International and Comparative Law (BIICL) argues that, post *Rock*, an NOM protects against unintended waiver. However, it also protects against intended waiver which must be a bad thing.

When colleagues told me about ‘tacit agreements’ I shuddered. I spent a lot of time trying to encourage formality, despite realising that formality can be seen as aggressive and legalistic. Macaulay cites one businessman saying that he does not want legal clauses ‘waved’ at him ‘I will not be treated as a criminal’^42^ and Clive Davies criticises overt formality suggesting that formality, as opposed to concentrating on a ‘fix it first and then sort the claims out later’ approach, (management) makes parties defensive.^43^

Let us consider a couple of plausible commercial scenarios.

- The tacit agreement referred to by one respondent has meant that no liquidated damages have been deducted for late delivery. The contract includes an NOM Clause. A new manager enters the scene and sees an opportunity to recover some of her company's pandemic losses by now deducting those liquidated damages, which were never waived expressly in writing. I am consulted. I tell her that what she proposes seems to be legally sound but morally dubious, noting that in *Rock,* senior management intervention overruled the deal made by a more junior employee. She presses ahead.
- A contract for off-site/remote software maintenance of an enterprise critical system is agreed upon during the pandemic. In putting commercials ‘out of the window’, the parties hold a Zoom ‘meeting’, agreeing that the rise in homeworking has, perhaps counter-intuitively, meant that some on-site work is required. The contract requires that the supplier deploys ‘Key’ personnel who must not be redeployed without the client's say-so. One of the Key technicians is clinically vulnerable and unable to work onsite. He is redeployed, with the client's knowledge but without the client's express permission. There is an NOM in the contract. As the parties try to unscramble the situation the client argues for a reduction in the rates paid for the Key technician's onsite replacement, on the basis that, should this not happen, they will consider triggering the termination provisions which expressly cover redeployment without consent. I am approached. I am aghast. I point them to Lord Sumption's poorly expressed carve out:

At the very least, (i) there would have to be some words or conduct unequivocally representing that the variation was valid notwithstanding its informality; and (ii) something more would be required for this purpose than the informal promise itself …^44^

It is worth wondering whether this is wide enough to protect the tenant doing gardening in lieu of rent.

I ask my colleagues whether, for example, they have paid invoices for the onsite work by the replacement technician. Have they held meetings with the replacement as if with the Key? I ask what the point is and am made to understand that there are budget and bonus issues at stake. I am not sympathetic, and advise them that the law might not be on their side as the replacement has been accepted onsite, and security checked, in lieu of the Key, and invoices have been paid without protest.^45^ That might be enough to represent ‘unequivocal’ conduct. They say that they were presented with a fait accompli and had no choice but to accept the replacement and they are going to deal with it in that way.

Back in my office, I hope that the next such call comes from my colleague in Singapore where the Court of Appeal has agreed with the Court of Appeal of England and Wales which in *Rock* took the position that an NOM creates only a rebuttable presumption.^46^ Perhaps the day will improve further and the next call might come from colleagues in New Zealand where the High Court appears to be following the pre-*Rock* England and Wales jurisprudence.^47^ In Australia, NOM clauses may be construed there as making it more difficult to conclude that conduct was intended to vary the contract.^48^ In Canada Courts have held that an oral variation may be valid despite an NOM clause, in one case where variations were agreed orally the Court saying that ‘where an owner has acquiesced in the provision of extras, it may be found to have made an implied promise to pay for them’.^49^ The position in New York is similar to the UKSC Rock position (but there are safeguards).^50^ In Texas, it seems that the position is broadly comparable to the pre-Supreme Court England and Wales position.^51^

Overall, however, I am considering whether to act as an adviser or as an executive. I have given my advice and must now consider whether it is compatible with the firm's ethical standards and whether the reputational consequences of this Court-facilitated opportunism are worth experiencing. It is not something that executives or advisors should have to ponder. Courts should support honest deal-makers and take the position that those who are performing a contract, if there is clear mutuality in the manner of performance, might have greater insight into party intention than third parties in sumptuous Courtrooms facing the Houses of Parliament.

## Reflections

Courts should not act as ‘destroyers of bargains’,^52^ but strive, like respondents, to make contracts work; and avoid putting ‘spanners in the works’ or ‘grit in the oil’.^53^ The recurring themes from respondents (and from the NRLA's respondents) are flexibility,^54^ and a sense of solidarity. There is no report of abandonment of deals, despite judicial spanners and grit.

Respondents appear to have filtered out the notional grit, the filter being, I would argue, the desire for and expectation of performance. There is some limited short-term evidence of taking advantage of the pandemic. The United Kingdom Government's advice in respect of contracting in mid-2020 included this:

Responsible and fair behaviour in contractual arrangements impacted by Covid19 will support the performance and viability of contracts.^55^

As we have seen it is questionable whether the Government's own behaviour meets that standard. See, for example, Mrs Justice O’Farrell ruling on Government conduct in one case ‘However, the Defendant's failure to consider any other research agency, by reference to experience, expertise, availability or capacity, would lead a fair-minded and informed observer to conclude that there was a real possibility, or a real danger, that the decision-maker was biased’.^56^ Respondents did not mention fairness but the thread which ran through their recollections was that they had tried to keep contracts alive, viable or not, and ensure that they could be performed, through pragmatic adjustments, such as ‘a mutual ‘tacit’ agreement that we won't invoke the specific dates’ (risky as we have seen).

In my comments to respondents, I said ‘Reading the responses was great fun. How I wish I was there! I can smell the early recognition that something big is happening. The informal email trails, the WhatsApp threads, the Corporate Instructions, departmental guidance, the lawyers’ opinions, the analysis of contracts, the formal email trails, the internal and external meetings, notices (formal and informal), the buds of disagreement. Then the diagnostics. What are the problems? How can we resolve them? Do we want to resolve them? The clamour for clarity; do our contracts cover us?, does our process allow me to deal with this?, who is likely to be difficult?, when do I make those calls? The new vocabulary (furlough, Pandemic, Wuhan), the corridor conversations, the rumour mill’.

Professor Coote describes the performance interest as meaning that what distinguishes a contract from other promises is that ‘it is intended to, and does in fact, confer on the promisee an enforceable legal right to have the promise performed’, implying, at least for the purposes of this article giving force to the deals made, the tacit agreements, the backroom nudges, the ‘work-arounds’, in the spirit of upholding the deal, removing the spanners, and of making performance a key dimension of adjudication,^57^ requiring hard-boiled contextualist interpretation, with Courts enforcing the actuality, the deal-making, the understandings, actual performance, and the expectations of constructive engagement and problem-solving.

## Notes

1. See Charles Haward Soper, *Commercial Expectations and Cooperation in Symbiotic Contracts – a Legal and Empirical Analysis* (London: Routledge, 2020), chap. 3.

2. Stewart Macaulay, “Non-Contractual Relations in Business: A Preliminary Study,” *American Sociological Review* 28, no. 1 (1963). Stewart Macaulay, “Non-Contractual Relations in Business: A Preliminary Study,” *American Sociological Review* 28, no. 1 (1963).

3. Roskill J in *Cehave v Bremer Handelsgesellschaft Mbh* [1975] 3 All ER 739 (1976). See Daniel Friedmann, “The Performance Interest in Contract Damages,” *LQR* 111 (1995): 629.

4. Kim Lewison, *The Interpretation of Contracts*, 3rd ed. (London: Sweet & Maxwell, 2004), 6.11.

5. Sir John Mummery, “Commercial Notions and Equitable Potions,” In *Commercial Law and Commercial Practice*, edited by Sarah Worthington (Oxford and Portland, Oregon: Hart Publishing, 2003), 37.

6. Russell J. Weintraub, “A Survey of Contract Practice and Policy,” Wisconsin Law Review, no. 1 (1992): 53. See also Hugh Beale and Tony Dugdale, “Contracts between Businessmen: Planning and the Use of Contractual Remedies,” *British Journal of Law and Society* 2, no. 1 (1975): 53 – “practice appeared to be quite close to the legal position”.

7. Lord Hoffmann's phrase in *A-G of Belize v Belize Telecom Ltd* [2009] UKPC 10 (2009). at [16].

8. *Marks and Spencer Plc v Bnp Paribas Securities Services Trust Company (Jersey) Limited* [2015] UKSC 72 (2015). at [21].

9. Matthew B. Miles and A. M. Huberman, *Qualitative Data Analysis: An Expanded Sourcebook*, Vol. 2 (Thousand Oaks, CA; London; Sage, 1994), 38.

10. See Norman K. Denzin and Yvonna S. Lincoln, *Collecting and Interpreting Qualitative Materials* (London: Sage, 1998), 204 – recommending “the qualitative researcher thinks purposively and conceptually about sampling.”

11. Bruce L. Berg, *Qualitative Research Methods for the Social Sciences*, Vol. 7 (Boston, MA;London: Ally & Bacon, 2009), 49–51, Earl R. Babbie, *Survey Research Methods*, 2nd ed. (Belmont, CA: Wadsworth Publishing, 1990), 97–98 and 99.

12. Mandy Burton, “Doing Empirical Research,” in *Research Methods in Law*, ed. Dawn Watkins and Mandy Burton (London: Routledge, 2012), 59.

13. Colin Robson, *Real World Research*, Vol. 3 (Chichester: Wiley, 2011), 275.

14. Berg, *Qualitative Research Methods for the Social Sciences*, vol. 7, 50–51.

15. Richard N. Landers and Tara S. Behrend, “An Inconvenient Truth: Arbitrary Distinctions between Organizational, Mechanical Turk, and Other Convenience Samples,” *Industrial and Organizational Psychology* 8, no. 2 (2015), 142-164. Alan Bryman, *Social Research Methods*, 4th ed. (Oxford: OUP, 2012), 191.

16. Bryman, *Social Research Methods*, 166–170.

17. Robson, *Real World Research*, vol. 3, 276.

18. Ibid.

19. Miles and Huberman, *Qualitative Data Analysis: An Expanded Sourcebook*, vol. 2, 27. Berg, *Qualitative Research Methods for the Social Sciences*, vol. 7, 8. “If humans are studied in a symbolically reduced, aggregated fashion”… conclusions may “fail to fit reality.”

20. Miles and Huberman, *Qualitative Data Analysis: An Expanded Sourcebook*, vol. 2, 267, Robson, *Real World Research*, vol. 3, 158 and 242–243, Denzin and Lincoln, *Collecting and Interpreting Qualitative Materials*, 199–200. Berg, *Qualitative Research Methods for the Social Sciences*, vol. 7, 7 – “Research literature continues to support Denzin's recommendation to triangulate.”

21. My thanks for this analysis to my colleague, ….

22. See Soper, *Commercial Expectations*, chap. 3.

23. See Julia Bear and Anita Woolley, “The Role of Gender in Team Collaboration and Performance.” *Interdisciplinary Science Reviews* 36 (2011), abstract. and Nicholas D. Wright et al., “Testosterone Disrupts Human Collaboration by Increasing Egocentric Choices.” *Proceedings of the Royal Society* 279 (2012).

24. Another possible area for research is the effect on working relationships created as women bear the brunt of additional housework during lockdown/ Linda Scott, “How Coronavirus Is Widening the UK Gender Pay Gap,” *The Guardian*, July 2020.

25. Eric Brunelle and Jo-Annie Fortin, “Distance Makes the Heart Grow Fonder: An Examination of Teleworkers’ and Office Workers’ Job Satisfaction through the Lens of Self-Determination Theory.” *SAGE Open* 11, no. 1 (2021).

26. Nick Clay, “Bright Lights Amid Gloomy Covid Outlook–Landlords Provide Helping Hand for Tenants.” National Rental Landords Association, https://www.nrla.org.uk/research/special-reports/cv-report22020.

27. Ben Chapman, “Illegal Evictions up 50 Per Cent Since Pandemic Began as Housing Experts Warn of ‘Massive Increase’ in Coming Months,” *The Independent* 2020.

28. Macaulay, “Non-Contractual Relations in Business: A Preliminary Study.”

29. National Audit Office, “Investigation into Government Procurement During the Covid-19 Pandemic,” *News release*, 2020. Elisabeth Mahase, “Covid-19: UK Government Faces Legal Action after Awarding £250m in PPE Contracts to Jewellery Company,” *BMJ* 2020 (2020). Gill Plimmer, Jim Pickard, and Anna Gross, “Shake-up for State Contracts after Criticism of Ppe Ties to Tories,” *Financial Times* 2020. For an update see – Revealed: Then there were 50! Government comes clean about three new “VIPs” – Good Law Project.

30. Beale and Dugdale, “Contracts between Businessmen: Planning and the Use of Contractual Remedies,” at 59.

31. Macaulay, “Non-Contractual Relations in Business: A Preliminary Study.”

32. Catherine Pédamon and Radosveta Vassileva, “Contractual Performance in Covid-19 Times: Does Anglo-French Legal History Repeat Itself?,” *European Review of Private Law* 29, no. 1 (2021).

33. Courts may affix the low bar of Braganza (*Braganza v Bp Shipping Ltd (the “British Unity”)* [2015] UKSC 17 (2015)) decision making duties of rationality on force majeure decision makers – see *Dwyer (Uk Franchising) Ltd v Fredbar Ltd* [2021] EWHC 1218 (Ch) (2021).

34. Paul Fisher, Eight Things to Know About the Art of Virtual Negotiation. In Oxford Answers (Oxford University Said Business School, 2020). https://www.sbs.ox.ac.uk/oxford-answers/eight-things-knowabout-art-virtual-negotiation:

35. FN 4 above.

36. *Rock Advertising Limited (Respondent) v Mwb Business Exchange Centres Limited (Appellant)*. [2018] UKSC 24 (2018). The Court has re-iterated the position in *Kabab-Ji Sal v Kout Food Group* [2021] UKSC 48 (2021).

37. *Prenn v Simmonds* [1971] 3 All ER 237 [1971] 1 WLR 1381 (1971), in which the House of Lords ruled that subsequent performance may not be taken into account in interpreting contracts. See Lewison, *The Interpretation of Contracts*, 3.19.

38. Lord Steyn in Worthington, *Commercial Law and Commercial Practice*, 128

39. Janet O'Sullivan, “Party-Agreed Formalities for Contractual Variation – a Rock of Sense in the Supreme Court?,” *Law Quarterly Review* 135 (2019).

40. Paul S. Davies, “Varying Contracts in the Supreme Court,” *Cambridge Law Journal* (2018).

41. https://www.biicl.org/documents/10695_revised_concept_note_2_on_the_effect_of_the_pandemic_on_commercial_contracts_bd.pdf at [72].

42. Macaulay, “Non-Contractual Relations in Business: A Preliminary Study.”

43. Clive Davies, “The Successful Management of Delay in It Outsourcing Contracts.” http://www.scl.org/site.aspx?i=ed39430 (2014).

44. *Rock Advertising Limited (Respondent) v Mwb Business Exchange Centres Limited (Appellant)*. at [16].

45. See *New Zealand Diving Equipment Ltd v Canterbury Pipelines Ltd* [1967] NZLR 961 (1967).

46. *Charles Lim Teng Siang v Hong Choon Hau* [2021] SGCA 43 (2021).

47. Marcus Roberts, “No Oral Modification Clauses in New Zealand – Now What Do We Do?,” *NZULR* 28 (2019).

48. *Mathews Capital Partners v Coal of Queensland Holdings* [2012] NSWSC 462 (2012). See *Liebe v Molloy* (1906) 4 CLR 347 – the High Court of Australia held that a clause stating that variations must be made in writing did not prevent an informal variation binding the parties at 353–355, followed in *GEC Marconi Systems v BHP* [2003] FCA 50.

49. *Ontario Inc. o/a Balkan Construction v Catan Canada Inc. et al*., 2013 ONSC 4727 (Ont. S.C.J.) at [14] also citing *Colautti Construction v City of Ottawa* (1984) 9 DLR (4th) 265. See https://www.martindale.com/construction/article_Perley-Robertson-Hill-McDougall-LLP-srl_2223756.htm – … if … the owner had waived [an NOM] because it had either given express or definitive instructions to do the extra work or it knew that the plaintiff was doing [it] and stood by and approved of what was being done and encouraged the plaintiff to do it, then the extra work was compensable.

50. https://www.nortonrosefulbright.com/en-us/knowledge/publications/faa7cea4/no-oral-modification-or-variation-clauses.

51. See e.g. *Shields Limited Partnership v Bradberry No. 15–0803, 2017 WL 2023602 (Tex. May 12, 2017).*

52. Lord Tomlin – *Hillas & Co Ltd v Arcos Ltd* (1932) 38 Com Cas 23 [1932] All ER Rep 494 (1932).

53. Sir Robert Goff, “Commercial Contracts and the Commercial Court,” *LMCLQ* (1984) 382 and 391.

54. See John W Barry et al., “Corporate Flexibility in a Time of Crisis.” https://papers.ssrn.com/sol3/papers.cfm?abstract_id=3778789 (2021), showing “how flexibility shapes companies’ responses to the onset of the COVID-19 crisis and drives longer-term changes in the corporate sector.”

55. Cabinet Office, “Guidance on Responsible Contractual Behaviour in the Performance and Enforcement of Contracts Impacted by the Covid-19 Emergency.” https://assets.publishing.service.gov.uk/government/uploads/system/uploads/attachment_data/file/899175/__Update_-_Covid-19_and_Responsible_Contractual_Behaviour_-_30_June__final_for_web_.pdf: UK Government, 2020. Cabinet Office guidance. See also the BIICL's guidelines at https://www.biicl.org/documents/10696_concept_note_3_guidelines_english_bd.pdf. These include acting fairly and responsibly, transparently, engagement in discussion, exploration of solutions.

56. *Good Law Project Ltd v Minister for the Cabinet Office* [2021] EWHC 1569 (TCC) (2021). [168].

57. Brian Coote, “The Performance Interest, Panatown, and the Problem of Loss,” *LQR* 117, no. January (2001): 82.
